# Determinants of persistent malaria transmission in the Arjo-Didessa farm area in Ethiopia

**DOI:** 10.4102/sajid.v39i1.623

**Published:** 2024-11-13

**Authors:** Hiwot S. Taffese, Sibusiso M. Zuma

**Affiliations:** 1 Department of Health Studies, College of Human Sciences, University of South Africa, Pretoria, South Africa

**Keywords:** malaria, determinants of malaria transmission, irrigation, mosquito, strategy, antimalarial intervention, behaviour

## Abstract

**Background:**

In tropical and subtropical areas of the world, malaria is still a serious public health concern. Activities related to agricultural development that involve irrigation schemes likely increase the risk of malaria in tropical and sub-Saharan African regions. Ethiopia is a sub-Saharan country where malaria is endemic.

**Objectives:**

The aim of the study was to investigate the determinants related to the persistence of malaria transmission in the Arjo-Didessa sugarcane farm area in southwest Ethiopia.

**Method:**

The study employed a quantitative survey design. Quantitative data were collected from 397 households using structured questionnaires. SPSS Statistics version 26 was used to analyse the data. Z- and Chi-square tests were applied, and the data were analysed using logistic regression.

**Results:**

The determinants that were identified included variation in land use, water management practices, socioeconomic status and knowledge about the use of anti-malaria treatment. These were shown to contribute to increased malaria transmission and the rise in malaria cases in agricultural areas.

**Conclusion:**

The effectiveness of malaria control in agricultural settings can be improved through free access to malaria testing and treatment as well as knowledge about anti-malaria treatment among the residents in agricultural areas.

**Contribution:**

The study revealed key determinants, including the promotion of free access to anti-malaria treatment, which should be considered for the effective management of malaria in agricultural areas.

## Introduction

In tropical and subtropical areas, malaria is a risk for a considerable proportion of the global population, imposing large health and socioeconomic burdens. The World Health Organization (WHO) estimated that about half of the global population (3.2 billion people) were at risk of malaria, with 229 million malaria cases and 409 000 malaria deaths registered in 2019. Africa was the most affected continent, accounting for 94% (215 million) of all cases, and the Asia Region accounted for about 3% of the burden of malaria cases globally.^1^

Endemic countries, including Ethiopia, have been implementing anti-malaria strategies for many years. Various challenges make it difficult to control malaria in developing countries. These include climate change, poverty, poor sanitation, weakened health systems, poor or a lack of disease surveillance tools, natural disasters, conflict, migration, ineffective antimalarial treatment and counterfeit medications.^2^

In Ethiopia, 75% of the landmass naturally facilitates malaria transmission. Malaria is highly seasonal, with severity changing according to altitudinal and climatic variations. Malaria-endemic areas are often described as those below 2000 m above sea level. Numerous residential areas in Ethiopia with micro-epidemiological settings suitable for malaria transmission are above this altitude. According to the Ethiopian government’s malaria programme, the National Malaria Strategic Plan 2021–2025 is aimed at reducing malaria morbidity and mortality by 50% from the 2020 baseline by the end of 2025. The strategic plan has an elimination-focussed goal to achieve zero indigenous malaria cases and to prevent the reintroduction of malaria in districts reporting zero indigenous malaria cases. The expectation is to eliminate indigenous malaria from the country by 2030.^3^

However, the risk of malaria transmission could be increased through agricultural irrigation development activities that provide favourable conditions for mosquito breeding habitats.^4^ High rates of mosquito bites occur both inside and outside agricultural workers’ residential housing.^5^ Irrigated agriculture activities alter the environment and promote the breeding of malaria vectors.^6^

Therefore, this study was aimed at analysing determinants associated with the persistence of malaria transmission in the Arjo-Didessa sugarcane development area in the Jimma Arjo District of Ethiopia.

## Research methods and design

A quantitative research survey design was applied.

### Setting

**FIGURE 1:**
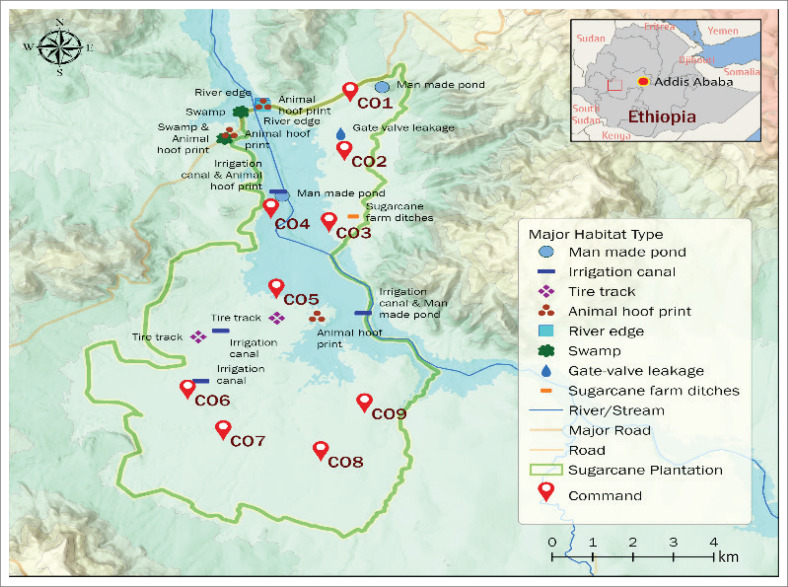
Map of the Arjo-Didessa sugarcane scheme.

The study was conducted at the Arjo-Didessa sugarcane irrigation site and its adjacent vicinity 395 km from Addis Ababa in the East Wollega Zone of Oromia Regional State. The Arjo-Didessa sugar factory is located at 8° 41’ 60’’ N, 36° 23’ 60’’ E (Figure 1). Altitude in the region ranges from 1600 m to 2500 m above sea level.^7^

### Study population

The population for this study resided in the villages adjacent to the Arjo-Didessa sugarcane irrigation scheme under the Jimma Arjo District administration.

### Data collection

Data were collected using a pre-coded structured questionnaire and Android tablet devices from 397 surveyed households (Online Appendix 1).

### Data analysis

The quantitative data were analysed using Statistical Package for Social Sciences software version 26. Both descriptive and inferential statistics were used in the analysis of the data. Z- and Chi-square tests were applied, and the data were analysed using logistic regression.

### Demographic profile of respondents

**FIGURE 2:**
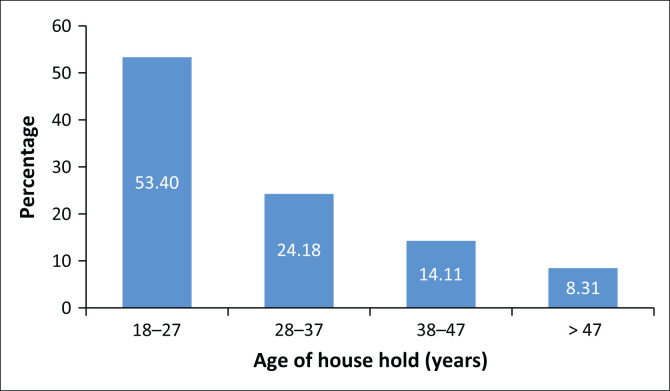
Respondents’ age categories (*N* = 397).

From the 403 respondents recruited for this study, 397 were interviewed face to face using the standardised questionnaire to gather the data. The data were collected from 217 female and 180 male study participants. Their mean age was 29.7 years (standard deviation [s.d.] = 11.2). The minimum age was 18 years and the maximum age was 82 years. The respondents’ ages were grouped into four categories, namely: 18–27 years, 28–37 years, 38–47 years and 48 years and above. Most respondents, 53.4% (*n* = 212), were in the age group 18–27 years, and 24.2% (*n* = 96) were in the age group 28–37 years (Figure 2). The respondents’ levels of education ranged from never having attended school to attending college or university. A small portion, 8.1% (*n* = 32), had at least a college or university degree and 48.4% (*n* = 192) had never attended school. Most respondents, 83.6% (*n* = 332), were agricultural workers; 16.4% (*n* = 65) were other professionals. Therefore, all the study respondents were employed. Of the respondents, 79.8% (*n* = 317) were married, 19.6% (*n* = 78) were never married and 0.6% (*n* = 2) were separated or widowed.

### Ethical considerations

Ethical approval was obtained from the University Ethics Committee (reference no.: 61983705_ CREC_CHS_2021), and gatekeeper permission was acquired from the regional health bureau. Furthermore, informed consent was obtained from respondents to ensure their voluntary participation, anonymity, and confidentiality, and assured that their responses would be used exclusively for research purposes and remain anonymous and confidential.

## Results

The main determinants of malaria include environmental factors, coverage of long-lasting insecticidal nets (LLINs), availability of health services, treatment-seeking behaviour, access to health facilities for malaria treatment, knowledge and awareness of malaria, gaps in malaria-related information, socioeconomic inequalities and behavioural aspects.

### Accessing health facilities for the treatment of malaria

The respondents who had experienced malaria during the last 12 months indicated that the main concerns for accessing health facilities were distance (94.4%) and poor health service (5.6%).

### Environmental, housing and economic status of respondents

Two hundred and ninety-six respondents (74.6%) had radios in their homes. Television was available to 66.5% (*n* = 264). Most (70.0%) respondents lived in houses built with corrugated iron sheets. Regarding housing floor material, 375 (94.5%) of the respondents had floors made from soil. The homes of 298 (75.1%) respondents had walls made from soil or wood. The respondents reported that 165 (41.6%), 84 (21.2%), 74 (18.6%) and 71 (17.9%) owned livestock such as goats, donkeys, sheep and horses or mules, respectively. Regarding types of fuel, almost all (97.0%) respondents used firewood or straw in their homes. The study revealed that indoor residual spraying (IRS) was an important malaria prevention method. Three hundred and fifty-two respondents (88.7%) stated that their houses had been sprayed with chemicals in the last 12 months. The homes of 64 (16.1%) of the respondents had not been plastered or painted in the last 12 months.

### Malaria prevention and long-lasting insecticidal net coverage

Most of the respondents (65.0%) were not using regular indoor insecticide spraying, while 35.0% did so to prevent mosquito bites. Three hundred and seventy-nine respondents (95.5%) did not regularly use coils. More than half (58.0%) reported that a lack of mosquito net availability was the main reason for not sleeping under nets.

### Health service availability and treatment-seeking behaviour

There was a significant association between family members and respondents experiencing malaria and having fever episodes (*p* = 0.023, *χ*^2^ = 7.96), as well as between malaria tests not carried out and the respondents’ lack of a budget or money (*p* = 0.002; *χ*^2^ = 4.57). This means that respondents or family members who have had fever episodes were more likely to have malaria than respondents or family members who had not experienced fever episodes. Also, respondents with no access to free malaria testing were more likely to have malaria.

### Knowledge of malaria among the respondents

Only 4.3% (38) of the respondents mentioned that malaria was associated with rain or stagnant water. Nearly half of the respondents, 43.6% (*n* = 390), thought that mosquitoes were the direct cause of malaria; 23.4% (*n* = 209) thought that sitting in the sun caused malaria.

Of the 397 respondents, 31.7% (*n* = 247) indicated that health workers were their source of malaria-related information. For 28% (*n* = 218), neighbours were a source of information while 27.1% (*n* = 211) indicated radios as their information source on malaria. Schools were a source of information for 10.8% (*n* = 85) of the respondents. Fifty-three per cent of respondents indicated that they had always known that malaria could be prevented with antimalarial drugs, while 33.6% knew about malaria prevention using spray. Only 28.8% of the respondents indicated that their only knowledge about malaria prevention was using bed nets. To the question ‘Will insecticide-treated nets still be effective against mosquitoes if you wash them?’, over half of the respondents agreed and 45.6% (*n* = 3) said that they did not wash dirty nets regularly.

The effectiveness of insecticide-treated nets (ITN) depends on regular and consistent use, and people’s perceptions about its usage. In this study, 70.0% of the respondents had a positive attitude about the benefits of sleeping under ITN and 28.8% understood their usage properly.

### Univariate and multivariate analysis of some determinates of malaria prevalence among the respondents

Suspected malaria cases revealed a statistically significant association with the respondent lifestyle. Those who had less than a university education were 1.4 times more likely to have malaria than those who had attended university (adjusted odds ratio [AOR] = 1.39; 95% confidence interval [CI]: 1.322–6.016).

## Discussion

The study found that socio-demographic factors including education and occupation were persistent determinants for malaria. The surrounding environment and type of housing also affect malaria control. The presence of stagnant water close to residential areas was associated with an increased risk of malaria. The results of this study further showed that people in the area have a high susceptibility to malaria and mosquito bites because of the area’s poor housing and living conditions. Houses on the farms were poorly constructed. Walls were made of grass or iron sheeting and wooden frames, and most had grass roofs. Housing structure has an impact on the use of ITNs. The observed shelter characteristics made it almost impossible to hang ITNs because of the lack of discrete sleeping areas in a packed multi-person shelter.

Our findings are consistent with others linking malaria to the type of human habitation and living environmental factors.^5,8,9,10,11^ Malaria prevention efforts are hampered by insufficient space inside the traditional huts, a small shelter constructed from local materials, for hanging a bed net.^12^

Respondents in this study exhibited appropriate knowledge and treatment-seeking habits. Community involvement is essential for interventions to be implemented successfully. The accessibility, acceptability and feasibility of IRS within the community are part of this. Indoor residual spraying was distributed equitably regardless of household wealth status. However, other studies reported that there were still some information gaps among respondents regarding the causes of malaria transmission.^13,14^

Access, acceptance and continuous application of preventative strategies are essential to prevent malaria. No matter how good a tool is, it will remain ineffective if communities do not interact with it or use it frequently.^15,16^

For ITNs to be effective, people must use them often and regularly.^13^ In our study, only 28.8% of respondents had a clear understanding of how to use ITNs, while 70.0% were positive about the benefits of sleeping under ITNs.

Most respondents described some of the causes and symptoms of malaria, which suggests considerable awareness of the disease’s symptoms. In support of this finding, a cross-sectional household survey from the Ha-Lambani hamlet of Vhembe District in Limpopo province of South Africa revealed that 259 of the 261 respondents accurately identified malaria symptoms, with at least one of the three most common symptoms (fever, chills and headache) being properly identified 99.2% of the time.^17^

In many agricultural areas, malaria is a serious public health concern, often increased by irrigation schemes.^17,18,19^ The respondents in a focal group interview had an insight about malaria and its associated risk in relation to agricultural practice.^20^ Another study found that there was a high level of risk perception about malaria and evidence from 16 African countries indicated the connection between agriculture and increased malaria risk.^21^

The study also found that understanding the burden of malaria and the impact of prevention interventions and environmental changes are critical for effective and efficient malaria control. The *woreda* [district] malaria community health worker emphasised the burden and difficulties brought on by the sugar fields, local geography, climatic variables and water accumulation in the surrounding area. The community health worker opinion was supported by other studies in Africa, where shifts in transmission risk for malaria were witnessed because of climate change: malaria transmission and seasonal suitability for malaria transmission were observed in climate change situations.^22^

## Conclusion

This study identified the primary determinants of malaria prevention and control to be factors related to inadequate bed net coverage, information gaps and socioeconomic inequalities in the study area. A comprehensive anti-malaria strategy and collaboration should be developed to prevent and control malaria in agricultural areas with irrigation schemes.
